# Health-care professionals' responsibility to patients' relatives in genetic medicine: a systematic review and synthesis of empirical research

**DOI:** 10.1038/gim.2015.72

**Published:** 2015-06-25

**Authors:** Sandi Dheensa, Angela Fenwick, Shiri Shkedi-Rafid, Gillian Crawford, Anneke Lucassen

**Affiliations:** 1 Clinical Ethics and Law, University of Southampton, Southampton, UK; 2 Wessex Clinical Genetics Service, University Hospitals Southampton NHS Foundation Trust, Southampton, UK

**Keywords:** duty to warn, ethical issues, genetic counseling, genetic testing, genomics

## Abstract

**Purpose:**

The extent of the responsibility of health-care professionals (HCPs) to ensure that patients' relatives are told of their risk is unclear. Current international guidelines take confidentiality to the individual patient as the default position, but some suggest that disclosure could be default and genetic information could be conceptualized as familial.

*Genet Med* **18** 4, 290–301.

**Methods:**

Our systematic review and synthesis of 17 studies explored the attitudes of HCPs, patients, and the public regarding the extent of HCPs' responsibility to relatives with respect to disclosure.

*Genet Med* **18** 4, 290–301.

**Results:**

Health-care professionals generally felt a responsibility to patients' relatives but perceived a variety of reasons why it would be difficult to act on this responsibility. Public/patient views were more wide-ranging. Participants identified several competing and overlapping arguments for and against HCP disclosure: guidelines do not permit/mandate it, privacy, medical benefit, impact on family dynamics, quality of communication, and respecting autonomy.

*Genet Med* **18** 4, 290–301.

**Conclusion:**

We argue that HCPs can sometimes share genetic information without breaching confidentiality and that they could factor into their considerations the potential harm to family dynamics of nondisclosure. However, we need more nuanced research about their responsibilities to relatives, particularly as genomic tests are used more frequently in clinical practice.

*Genet Med* **18** 4, 290–301.

Health-care professionals (HCPs) involved in genetic/genomic medicine will often become aware that patients have relatives who are at risk. HCPs will generally encourage patients to share relevant genetic information, especially if risk-reducing intervention is available.^1^ But to what extent do HCPs have a responsibility to ensure that relatives are informed?

Family communication studies have shown most patients tell—or intend to tell—at-risk relatives (herein referred to as “relatives”), but some do not, for reasons including guilt and fearing blame, poor relationships, difficulty finding the right time, and perceived inability to communicate accurately.^2,3,4,5^ Moreover, research suggests that patients perceive more clearly defined responsibilities to tell first-degree relatives than wider family, and that relationship closeness, rather than intervention availability, often drives communication.^6,7^

Patients may also be unsure of HCPs' responsibility. Of patients with Lynch syndrome, 56% (45/80) thought that HCPs, rather than themselves, should inform relatives, and, consequently, 12% failed to communicate risk.^7^ Some patients have reported that HCPs did not tell them sharing was important or prompt them to share.^8^ These findings are important because noncommunication or delayed communication can have serious ramifications—a study of 30 parents with Lynch syndrome showed that several postponed communication with offspring, some of whom later developed a preventable cancer.^9^

When patients have refused to communicate to relatives or when HCPs are unsure whether they have done so, HCPs face conflicting duties to respect patient confidentiality, privacy, and autonomy but also prevent possible harm and promote benefit to relatives. Internationally, guidelines assert that confidentiality is not absolute and give HCPs the “privilege” of breaching it under certain circumstances.^10^ UK General Medical Council guidelines^11^ state that HCPs can share information without explicit patient consent when the benefit of disclosure outweighs the interest of the public and the patient in keeping the information confidential and when nondisclosure puts others at risk of death or serious harm.

Guidelines focus mainly on situations when patients refuse consent. What HCPs can and should do when they are simply unsure whether communication has happened is unclear. Guidelines also take individual confidentiality as the default position; breaches are acceptable only under exceptional circumstances. Parker and Lucassen^12^ have argued that HCPs could instead err on the side of disclosure rather than confidence. They propose a “joint account” model of confidentiality, in which patients' clinical information (e.g., their identity and test result/diagnosis) is conceptualized as confidential, but genetic information (e.g., the mutation) is conceptualized as familial information, the sharing of which would not be a breach of confidence. They urge HCPs to take disclosure as the default option and identify reasons that would justify excluding other “account holders” from what is essentially their information. This model is the basis of current British Society for Genetic Medicine confidentiality guidelines.^13^ Similarly to Parker and Lucassen,^12^ Gilbar^14^ argues that HCPs should move away from individualistic notions of autonomy and toward a relational approach. This approach emphasizes that people develop autonomy through social embededness and engagement with others, and that people have an interest in maintaining familial relationships.

Findings from interventional studies show that when HCPs take active steps to ensure that information has been communicated (e.g., by writing directly to relatives, inviting them to counseling and educational sessions, or confirming at follow-up appointments that patients have told relatives), testing uptake among relatives at risk for Lynch syndrome and hereditary breast/ovarian cancer has significantly increased.^15,16,17^ Other studies, with relatives contacted through cancer registries, have shown that they appreciate such contact.^18,19^

Little research has been done on the attitudes of HCPs, patients, and the public on the responsibility of HCPs to ensure that relatives, including those who are not their patients, are informed. To identify consensus and contention regarding the issue, a systematic analysis of the research conducted thus far is needed. The need is particularly pressing, first, because array comparative genomic hybridization, whole-exome sequencing, and whole-genome sequencing are rapidly replacing more targeted genetic testing in the clinic. Because these tests produce information of greater volume and complexity, findings in patients that also predict ill health in their relatives are likely to increase.^20^ Second, HCPs whose specialties lie outside genetics and who will not necessarily have experience managing families are beginning to incorporate genetic and genomic tests into practice.^20^ We therefore conducted a systematic review and mixed-methods research synthesis about perceptions of HCPs' responsibility to relatives and views on the role of HCPs in disclosure.

## Materials and Methods

### Search strategy and study appraisal

**Figure 1:**
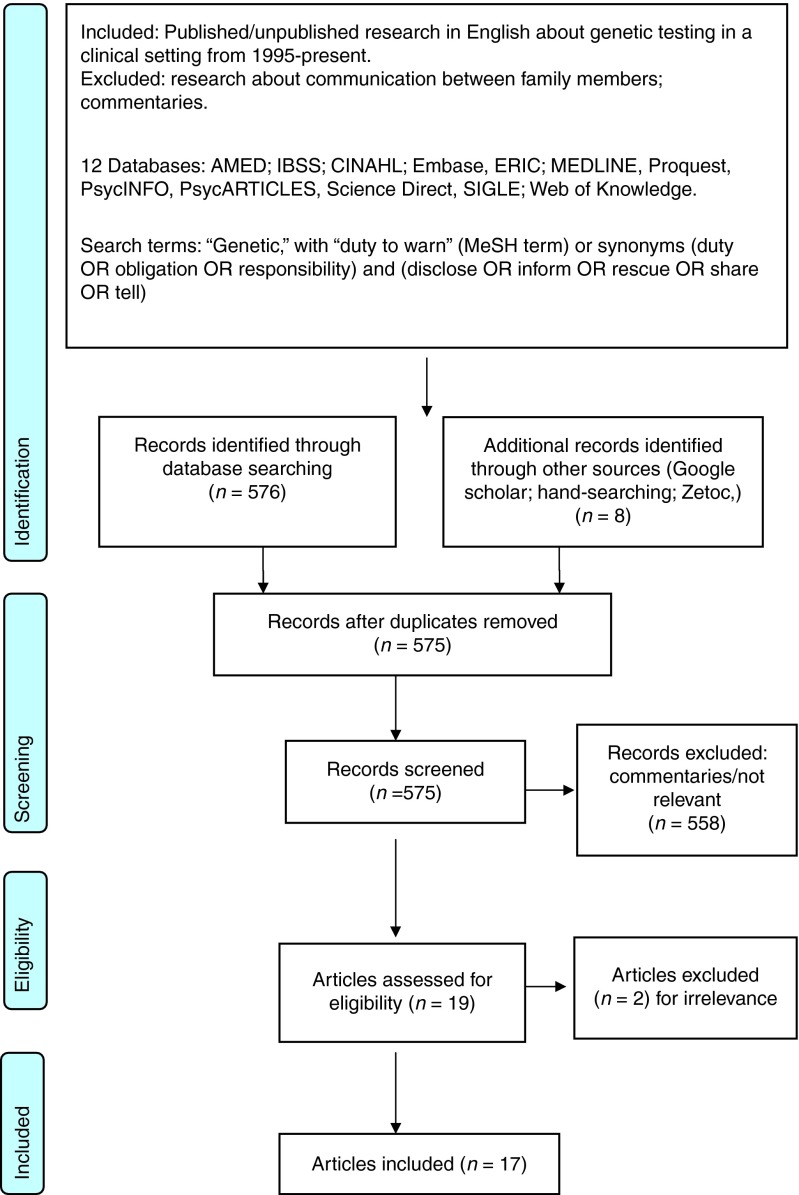
**PRISMA flow diagram.**

Using systematic review guidelines,^21^ we searched for empirical research studies using the databases, keywords, and inclusion/exclusion criteria shown in **Figure 1**. We excluded research exploring communication between family members because much has already been written about the topic. The 17 papers thus retrieved (HCPs, *n* = 8; HCP/patients, *n* = 1; patients, *n* = 6; the public, *n* = 2) are detailed in **Table 1**. Three with patients^22,23,24^ had only small relevant sections. Most studies focused on inherited cancer risks, often classified as actionable because of surveillance, chemoprevention, or risk-reducing surgical options.

S.D., A.F. and A.L. independently appraised the quality of each paper^25^ and agreed on its inclusion. A general limitation was that study authors often used hypothetical scenarios to gather views but these might not represent real-life actions. Another limitation was that, except for a few,^26,27,28^ most asked about disclosure when patients explicitly refused consent rather than when HCPs were unsure whether patients had disclosed, and so our synthesis focuses more on the former. A third limitation was that many studies used surveys, which provide useful generalizable data but do not always allow participants to discuss complex views in detail.

### Heterogeneity in studies

Our study population was heterogeneous, originating from 10 countries (United States, United Kingdom, Australia, Israel, Norway, Sweden, Canada, Slovenia, Turkey, and the Netherlands) and including a variety of participant groups. For example, four HCP studies involved HCPs working outside genetics. Participants' views may have been influenced by differences in culture and practice between and within these groups/countries, e.g., health-care systems (insurance-based versus free-at-the-point-of-use) and social attitudes toward genetic conditions.^29^ Across the 10 countries, guidelines/laws are generally similar in permitting disclosure without consent in narrow circumstances (see **Supplementary Table S1** online). Notable differences are that some UK guidelines encourage HCPs to consider disclosure over confidence when appropriate,^13^ Israeli law requires hospital ethics committees to approve disclosure,^30^ and Norwegian law completely disallows disclosure without patient consent.^31^ Guidelines/laws provide some context, but they are unlikely to completely shape professional practice norms or participants' views. We attempt to make differentiations when possible but, because of this heterogeneity, as well as the relatively small number of retrieved studies, the synthesis findings are tentative.

### Data extraction and analysis

To create the synthesis, we, as per previous research,^32^ thematically analyzed findings from each study. To do so, we listed each relevant concept discussed in each study (omitting, for example, views about disclosure to employers) and grouped similar concepts to create a thematic structure. S.D., A.F. and A.L. independently reviewed themes and scrutinized studies to explain variations and integrate “deviant cases” (those not fitting the general structure). Each heading below represents a theme. **Table 2** shows which studies raised each theme.

## Results

### Terminology and definitions varied

“Nondisclosure” was conceptualized by Clarke et al. (United Kingdom/Australia)^33^ as the situation of a HCP thinking that a patient should disclose information to relatives (because failure to do so could lead to harm) but they seemed unlikely to disclose. Two US studies similarly described it as a patient's refusal to notify relatives.^34,35^

Between and within studies, authors used varying phrases, including “responsibility to warn,”^22,39^ “duty to warn,”^26,27,28,31,34,35,36,37,38,39^“duty to inform,”^28,30,40^ “obligation to disclose,”^33,37^ “obligation to share,”^34^ and HCPs' “right to disclose to relatives.”^31^ “Consent” and “permission” were also used interchangeably. There were no obvious differences in their application, but the variety of terms suggests possible limitations in how HCPs/researchers communicate with one another. None of the studies specified what “warning” and other nomenclature meant in practical terms. We therefore use “HCP disclosure” here to capture any way in which HCPs might be practically involved in informing relatives (e.g., via direct contact or other genetics centers or relatives' general practitioner).

### Patients/public: wide-ranging views

Except for one,^31^ studies were framed such that participants probably took patients' rather than relatives' perspectives and considered that HCPs would be sharing *their* information without their consent.

In approximately half the studies, patients had unfavorable views of HCPs sharing information without consent. Patients in Israel^30^ undergoing testing for hereditary cancers and prenatal risks (*n* = 564) on average thought that HCPs had no right to disclose genetic information to relatives without consent. Patients in that study thought genetic information was familial but also personal. In fact, a minority (5.3%, *n* = 30) said they did not intend to share genetic tests results with family. However, no distinction was made between HCPs sharing general genetic information (e.g., the mutation) versus more personal information (e.g., patients' diagnosis or in whom the risk had been found).

Plantinga et al.^24^ also reported unfavorable views among 397 patients (United States) affected by breast/colon cancer, sickle cell disease, or cystic fibrosis. Only approximately 30% said they “would not mind” HCPs giving specific information (“medical information about me”) to relatives without permission and approximately 50% thought HCPs should be punished for doing so. In a US study with 238 women,^22^ when asked whether HCPs should be able to “disclose the results of genetic tests for hereditary breast/ovarian cancer” to immediate relatives without written consent, 87% thought they should not. Another US study^40^ found similar views among the public (*n* = 200): less than one-quarter thought HCPs should seek out and inform relatives about risk and prevention options against patients' wishes across three scenarios (Huntington disease (HD), breast cancer, and colon cancer).

Patients had somewhat more favorable views about HCP disclosure in three other studies. Of 105 patients (Canada),^27^ most (55.8%) wanted HCPs to tell at-risk relatives without their permission. However, others said they would not have sought genetic counseling if they knew HCPs could inform without permission. Pentz et al. (United States)^28^ found that 76% of 80 patients said “Yes” when asked whether it was permissible for HCPs to inform relatives. Very few patients added caveats such as “with permission.” They also thought patients and HCPs had a duty to inform relatives. In another study, with 104 patients (Turkey),^39^ who were given a hypothetical scenario, most (80.8%) disagreed that HCPs should respect a patients' wishes to withhold information about a balanced translocation, and most agreed that HCPs should tell the spouse (84.6%) and siblings (52.9%)—who were all at reproductive age and wanting children.

### From the relative's perspective

Only one study^31^ explicitly asked participants to take the relative's perspective (i.e., consider that a HCP is sharing information with them rather than about them). Slightly less than half of the 1,200 participants (Swedish public) said they would want a HCP to inform them against the related patient's wishes of a fatal, nontreatable condition with full penetrance, and slightly more than half would want the HCP to do so if the condition were nonfatal and treatable with 50% penetrance. The authors also asked 607 students in Norway, where the law forbids disclosure without consent. Possibly because of the law, a smaller but still substantial proportion (approximately 20%) wanted HCPs to inform them of each type of condition. Notably, in another study (United Kingdom),^23^ a patient who found out about his HD risk when a sibling was diagnosed was angry because distant relatives already knew about the risk. He thought that health services should have mechanisms to warn people but recognized that implementation would be difficult.

### HCPs generally felt some responsibility to relatives

In two qualitative studies (the Netherlands and the United Kingdom),^26,41^ HCPs briefly discussed having a moral responsibility to relatives. More specific data came from a study with genetic counselors and clinical geneticists (United States),^34,35^ in which 63% (161/257) and 69% (143/206), respectively, said they perceived an obligation.

Two studies with nongenetic HCPs also showed that they felt a responsibility to patients' relatives, including family physicians (United States)^36^ who, on average, did not think they had a legal “duty to contact” but would be morally justified in doing so, particularly with treatable conditions. Of 155 pediatricians and obstetricians/gynecologists in Turkey who considered the scenario of a patient refusing to tell his siblings and wife about his balanced translocation diagnosis, most (78.5%) agreed that they should inform the spouse.^39^ A smaller majority (41.2%) agreed that HCPs should tell siblings, with the remainder divided evenly between “disagree” and “neutral.” The majority (58.3%) disagreed that they should respect the patient's decision. Family doctors in Slovenia^38^ considered the scenario of a 35-year-old refusing to tell his 30-year-old brother (also their patient) about a hypertrophic obstructive cardiomyopathy risk. Of 271 doctors, 73.9% would directly inform and only 34.1% would respect patient wishes. In both studies, more occupational experience significantly predicted the decision to inform. Occupation could also explain why more clinical geneticists than genetic counselors stated they would warn in the US studies.^34,35^

Fewer (but still a substantial number of) nurses working outside genetics^37^ felt they should “take steps to inform” relatives if a patient refused to do so (17.8% fragile X; 30.1% breast cancer; 24.7% HD). However, for each condition, approximately 85% also agreed that they should respect confidentiality—meaning that some answered “agree” to both questions, a contradiction that the authors did not explain. 

### HCPs only sometimes acted on this responsibility

Four studies discussed nondisclosure frequency (albeit relying on HCPs' subjective reports). A prospective survey^33^ across 12 UK and 2 Australian centers showed only 65 nondisclosures of nearly 40,000 genetic clinic consultations (<1%) in 1 year. Most were for HD, translocations, and hereditary breast/ovarian cancer or colorectal cancer; 39 were to adult children, 22 to siblings, and 4 to current or former partners at risk of having affected children. No HCPs disclosed without permission, but the authors did not report the reasons. In another UK study,^41^ genetic HCPs were not specifically asked about disclosure dilemmas, but two reported sharing genetic information with patients' offspring without consent.

Two US studies reported higher nondisclosure rates. In one,^35^ 60% (123/206) of clinical geneticists reported patients who refused to disclose at least once (mostly translocations and “genetic syndromes”). Some individuals reported more than 16 instances of nondisclosure. Unlike in the UK/Australia study,^33^ 25% (31/123) considered informing relatives without explicit consent, and 4 of the 31 did so; how they did so was unreported. In the other US study^34^ with genetic counselors, 46% (119/250) reported experiencing refusals (mostly involving translocations and inherited cancer risks). Twenty-two (18%) considered disclosing without consent, but only one did, because the relative was her patient and the result was negative. Neither US study reported which relatives patients refused to tell or the timeframes in which these nondisclosures took place. 

### Arguments for acting/not acting on responsibility

Participants expressed several overlapping ethical, social, and practice-related arguments for and against HCPs acting on their responsibility (e.g., by sharing information). Some arguments also help to explain why some HCPs did not perceive a responsibility in the first place. We have listed arguments in decreasing order of the number of studies that raised them (except the first, which we place there because it encompasses other arguments). In parentheses, we show which group(s) discussed each.

1. (Mis)interpretations of guidelines and the law (HCPs/patients)

Five studies reported that participants had misconceptions about guidelines and/or the law, and some authors discussed how awareness, or lack thereof, possibly influenced views. For example, Stol et al.^26^ thought misinterpretations would prevent HCPs taking action when they could. In their study, some clinical geneticists believed the population-screening act disallowed unsolicited contact with patients' relatives. However, clinical genetic practice falls outside this legislation; HCPs can legally inform relatives of risks without patient consent. From the opposite perspective, Barnoy and Tabak (Israel)^37^ thought that a lack of understanding of Israeli law (which takes a relatively individualist stance) explained why some nurses said they would take steps to inform relatives if patients refused.

In the United States, 24% (61/253) of genetic counselors^34^ and 66% (146/206) of clinical geneticists^35^ erroneously believed that federal guidelines regulate disclosure. Also incorrectly, 35% (89/254) of genetic counselors and 29% (60/206) of clinical geneticists thought the National Society of Genetic Counsellors and the American College for Medical Genetics and Genomics, respectively, have specific guidelines for disclosure without consent. Less than half of both groups (44% of genetic counselors; 38% of clinical geneticists) were aware of American Society of Human Genetics and Genomics guidelines, which permit disclosure in certain circumstances. The authors suggest that this knowledge dearth might explain why so few disclosed in situations where guidelines would permit disclosure, but also that those aware might not feel obliged to warn because guidelines do not provide specific mandates to do so. Stol et al.^26^ also cited the lack of specific legislation or guidance as a reason for geneticists' confusion. Liability was a related concern in the United States and the United Kingdom,^34,35,41^ and some HCPs in the United Kingdom worried that certain situations did not meet the General Medical Council's “serious harm” criteria.^11^

Patients also held misconceptions—one thought that disclosure without consent would violate Canada's “privacy of information protection act” (no such act exists).^27^ By contrast, patients in one US study^28^ said that it was important that HCPs disclose despite concerns about liability.

2. Privacy

2i. Disclosure puts privacy in jeopardy (HCPs/patients)

Clinical geneticists and genetic counselors (United Kingdom and United States) ^34,35,41^ were reluctant to disclose to relatives because they thought patients saw information as private and because laws protected privacy. Indeed, the clinical geneticists who faced disclosure dilemmas ranked respect for patient confidentiality as the most important consideration in decisions not to disclose. Similarly, most (62%) pediatricians and obstetricians/gynecologists (Turkey)^39^ said they thought genetic information belongs to individuals rather than family or humanity in general. Clinical geneticists (United States)^35^ also thought that protecting privacy is integral to doctor–patient relationships. A few patients (Canada)^27^ agreed that HCPs could damage these relationships by disclosing. Patients corroborated that they saw genetic information as private: it was the main reason that 22/30 patients in Israel^30^ intended not to tell relatives of prenatal/hereditary cancer risks (although the perceived meaning of genetic information was unclear). In addition to patient privacy, clinical geneticists in the Netherlands^26^ worried about invading *relatives*' privacy by disclosing, and one patient in Canada^27^ also raised this issue. They thought some relatives might not want to know about risk and patients would be better at making these distinctions.

2ii. Privacy is easily trumped (HCPs/patients)

While considering genetic information as being private to individuals, patients in Canada and the United States^27,28^ also thought that privacy should not trump others' right to know relevant information, particularly when it could prevent illness or death, and when breaching a single person's confidentiality could help many people. This view reflected a utilitarian approach, i.e., maximizing benefit.

Some patients in Turkey^39^ did not think of genetic information as private to the individual: of 104, most (49%) thought it belongs to “the family,” aligning with the joint-account model, and some (12%) thought it belongs to “humanity.” Fewer (39%) answered “individuals.” Patients and HCPs who thought that information is familial were significantly more likely to agree that doctors should share if patients refuse. In fact, at least 60.5% of those who considered genetic information individual also thought doctors should share. The fact that the family unit is “the most important primary and intimate unit”^39^ in Turkish society may explain why genetic information was considered familial in this study, but discerning the role of culture was difficult because other authors did not ask similar questions. Nevertheless, some patients in the United States^28^ made more specific distinctions between individual and familial information. They thought relatives have a right to know general information (the “genetic alteration,” as phrased by one patient) and sharing it would not constitute a breach, but they also thought relatives do not have a right to know specific patient results or the patient's identity. They said HCPs can be trusted to make this distinction and share accordingly but that relatives might not always do so (e.g., would probably reveal patient identities), thus thinking that HCP disclosure would better protect privacy.

3. Medical benefit

3i. Medical benefit to relatives outweighs harm of breach (HCPs/patients)

In US studies, clinical geneticists^34^ and genetic counselors^35^ who experienced disclosure dilemmas rated medical benefit as highly influential in decision making, specifically with respect to the relative's age, the risk magnitude, treatability, manageability, and early monitoring.

Treatability and relative's age were also important considerations for nongenetic HCPs. A higher proportion of nurses in Israel^37^ said that they would take steps to inform relatives about hereditary breast cancer risks (where intervention is available) than about fragile X or HD. Given a scenario involving nondisclosure by a man to his children, 165 family physicians (United States)^36^ were, on average, unsure whether children should be informed about the risk of a potentially fatal condition but tended to favor disclosure more—particularly with older children (22-year-olds versus 17-year-olds)—if the condition was treatable rather than nontreatable.

Two studies (among family physicians in Slovenia^38^ and pediatricians and obstetricians/gynecologists in Turkey^39^) did not explicitly discuss medical benefit, but both used dilemma scenarios in which the conditions had available interventions. This possibly influenced the majority decision to disclose observed in these studies.

3ii. Determining medical benefit is complex (HCPs)

A minority of HCPs queried the “medical benefit” justification. Clinical geneticists in the Netherlands^26^ questioned whether interventions were beneficial. They explained that hereditary cancer screening had “unproven qualities” and preventative options were “extremely drastic, if not draconian.” They nevertheless thought it was “fair to inform people” but implied that HCPs should make these limitations explicit. A genetic consultant in a UK study^41^ noted that interventions need not be medical. She said that knowing was “almost like having an intervention” and did not think that “having an intervention makes such a difference.” On this basis, she told the symptomatic offspring of a patient with HD (in the absence of familial communication) that they were at risk. Notably, in a study with the Swedish public,^31^ more than half said they would want HCPs to inform them, even against relatives' wishes, about fatal conditions with no available intervention—although these hypothetical views might not match actual decisions.

4. Family dynamics

4i. HCP disclosure can damage family dynamics (HCPs)

Genetic counselors (United States)^34^ who had faced disclosure dilemmas considered patient–relative relationships and patients' emotional reactions two of the most influential considerations in their decisions about whether to disclose. In comparison, clinical geneticists (United States)^35^ who faced disclosure dilemmas focused less on social/emotional issues but still rated patient–relative relationships as the most important nonmedical consideration. These HCPs as well as those in a study by Clarke et al. (United Kingdom/Australia)^33^ perceived patient noncommunications as being due mostly to familial issues (estrangement, difficult relationships, and protecting relatives). Indeed, patients in a Canadian study^27^ gave such reasons for noncommunication. HCPs might have felt worried that they could exacerbate these perceived problems by disclosing information.

4ii. HCP disclosure can help family relationships (HCPs)

HCPs in a UK study^41^ argued that secrets could cause resentment and, by informing relatives when patients refuse, they could encourage open communication within families and consequently help relatives maintain close relationships and support one another, which the author argued is particularly important when facing difficult medical decisions. This view, reflecting a relational approach to autonomy, may have been influenced by the guidelines of the British Society for Genetic Medicine,^13^ which encourage HCPs to take disclosure rather than confidence as the default position.

5. HCPs can communicate better than patients and relatives (HCPs and patients)

Although patients, in general, said they preferred that relatives, rather than HCPs, tell them about risk, some argued that HCPs would be more reliable communicators. For example, some said HCPs would communicate facts more accurately and could more easily surmount obstacles of geographical distance, “social/emotional distance,” and “fragile family relationships.”^28^

In studies in the United States^35^ and the Netherlands,^26^ clinical geneticists said that patients might have inadequate knowledge about who was at risk. In the latter study, however, they also thought that patients would be better able to consider the possible psychological harms of disclosure as well as to judge which relatives would benefit from knowing and which might not cope well emotionally (although this might be the case only when the patient wants to disclose and the family are close). HCPs also thought it would be too problematic to disclose directly to patients' relatives because of lack of contact details and insufficient time, money, and staff.

6. Respecting (individual) autonomy (HCPs)

Clinical geneticists in the Netherlands^26^ worried that they would appear directive and relatives would feel pressured into being tested if they, rather than other family members, told them about their risk and options. They argued that they should remain nondirective, e.g., about whether patients should tell relatives, and whether relatives should undergo testing. However, others said patients could also pressure relatives into testing, highlighting the difficulty in ensuring that those being tested are making autonomous decisions.

### Conclusions in the reviewed studies

Only four papers offered a conclusion as to what HCPs should do when facing disclosure dilemmas: three (from within and outside genetics, in Turkey, the United Kingdom, and the Netherlands)^26,39,41^ suggested that HCPs could justifiably inform relatives when patients fail to do so, and one (outside genetics, in Israel)^37^ suggested that HCPs should continue to emphasize patient autonomy and privacy. Most (*n* = 8) concluded that guidelines should be refined to better address dilemmas,^22,30,34,35,36,38,39,40^ although two advocated leaving room for interpretation so that HCPs could treat dilemmas on a case-by-case, rather than routine, basis.^23,33^

## Discussion

**Figure 2:**
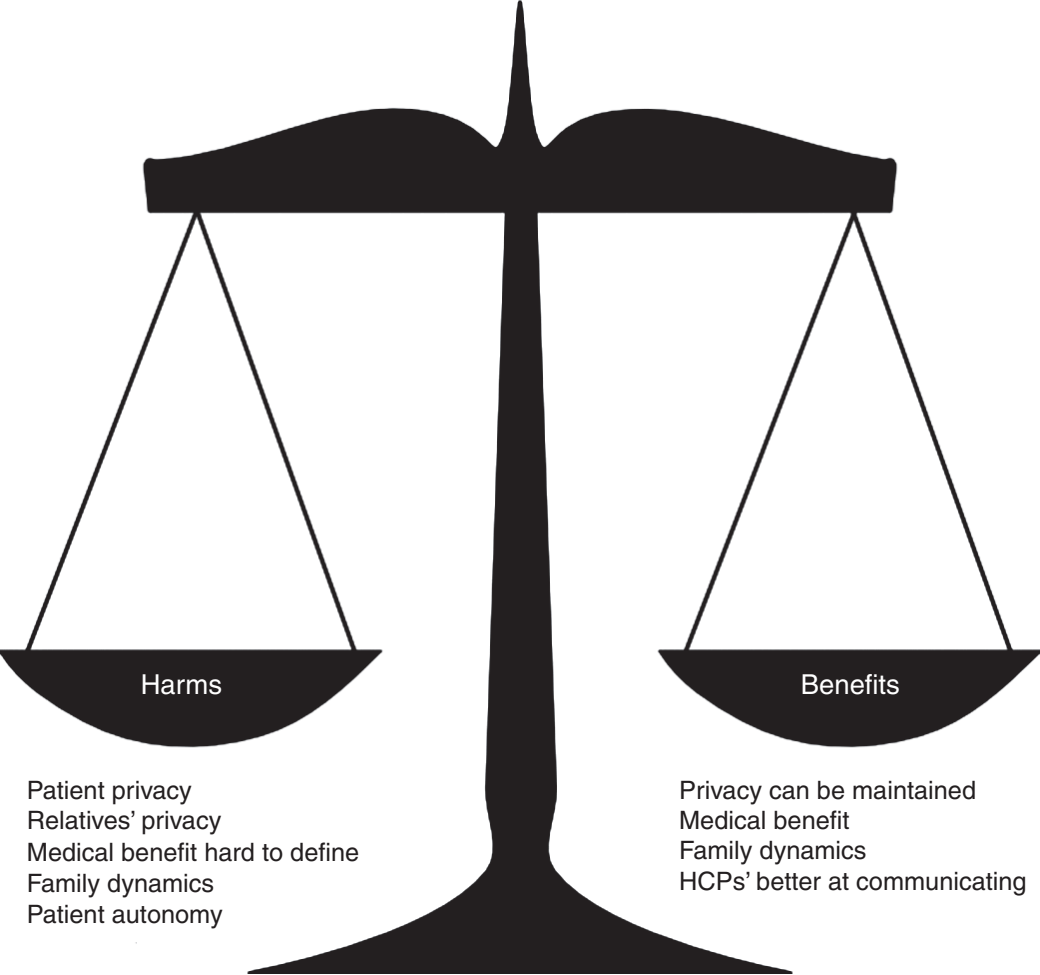
**Summary of arguments.** HCP, health-care professional.

This synthesis provides insight into perspectives on HCPs' responsibility to patients' relatives and HCP disclosure. HCPs generally felt responsible for relatives but perceived several obstacles to acting on this responsibility. The public and patients regarded HCP disclosure as unfavorable in approximately half the studies. In six competing and overlapping arguments, participants discussed the harms and benefits of disclosure by HCPs (summarized in **Figure 2**). Most arguments were drawn from HCP studies: public/patient views were underexplored. Limitations of the reviewed studies (including heterogeneity in populations and use of hypothetical scenarios) make our synthesis tentative, and its findings are in need of further consideration and research. Therefore, we turn to the wider literature to examine barriers to disclosure that HCPs might overcome and which arguments need more interrogation.

Patients in our review thought that HCPs should protect patient privacy and maintain confidential doctor–patient relationships by not disclosing. They also said that unsolicited contact with relatives could invade their privacy. However, relatives directly contacted by HCPs through registries have not felt that HCPs invaded their privacy and, although anxious about the news, were pleased to be informed.^15,19^ Indeed, in our review, participants generally thought privacy was less important than the opportunity to prevent serious illness or death. Moreover, when patients were asked to consider privacy in more detail, they said “genetic information” was familial and that HCPs could protect privacy and confidentiality by sharing de-identified information. These nuanced views were explored in only two studies and merit further research, particularly because “genetic information” can be an ambiguous phrase and it is not clear what “levels” of information (e.g., general information about familial risk/specific mutation/patient's individual result) participants considered private. More research is also needed about privacy in the genomic medicine context, in which most studies have focused on concerns about researchers, insurers, and employers accessing sequence data, rather than patients' willingness to share genomic information with family.^42^ However, in one study with 563 parents of children undergoing sequencing, only 5.2% thought that results should not be shared with relatives, and 93 and 88% perceived a right to be informed if a sibling's genome sequence showed a familial risk of a serious treatable or nontreatable condition, respectively,^43^ suggesting favorable views of information sharing.

Family dynamics was another important consideration for HCPs, who were concerned that HCP disclosure could have a negative impact. In the wider literature, patients telling relatives about genetic risk/diagnoses reported feeling blamed or that relationships had become hostile.^44^ The same might occur if HCPs shared information about a patient whose identity was obvious. However, in some cases HCP disclosure when a patient has refused might be better for family dynamics than respecting patient wishes not to share, given that secrets and disclosure delays were shown in our review,^23,41^ as well as in other research,^29,44,45^ to damage relationships and cause disappointment and resentment. Relatives unaware of risk might also eventually question why HCPs had not told them.^46^ By contrast, good communication about genetic risks could bring families closer together.^44^ Again, HCPs sharing general rather than specific information might protect individual patients from blame, although patient identity might sometimes be obvious.

A related concern was that HCPs did not want to be directive in telling patients to disclose, but it has been suggested that nondirectiveness is not appropriate for all aspects of genetic consultations/counseling.^47^ Arguably, accepting patients' decisions not to tell relatives is based on an overly simplistic understanding of what an “autonomous decision” is, and HCPs should direct patients away from decisions they might later regret or that put relatives at harm.^48^

Several studies reported that HCPs lacked awareness of, or had misinterpreted, guidelines or the law. The influence of guidelines was ambiguous. HCPs aware of them might be either more likely to disclose (because they know it is permitted) or less likely to disclose (because they worry the situation does not fit the criteria for which disclosure is permitted or because guidelines do not mandate disclose). Eight papers concluded that HCPs need clearer guidelines and policies on handling disclosure dilemmas and appropriate sharing, although our synthesis suggests that this may not help, because HCPs do not always know what the guidelines or relevant law says. Professional practice norms and personal values may also have influenced the attitudes of HCPs and are worth delineating in future research.

None of the reviewed studies addressed what action HCPs could take if erring on the side of disclosure and whether such an approach would be practical, especially when resources are constrained and little or no reimbursement is made for time spent counseling patients' relatives.^49^ Resources are likely to suffer additional strain as HCPs more frequently use whole-exome sequencing/whole-genome sequencing; although sequencing cost is decreasing, interpretation remains difficult and expensive.^50^

Involving family members, at least in the initial stages of clinical integration of genomic medicine, will be crucial. Uncertain results from a genome sequence will sometimes require HCPs to test relatives to clarify meaning. Moreover, incidental findings (e.g., *BRCA1* gene mutation in a boy with developmental delay) might be more relevant to relatives' health than to patients' health.^51^ These issues reinforce the importance of exploring HCPs' responsibility to relatives and whether, when, and to whom HCPs should disclose information.

### Recommendations

Although further research is needed, we recommend that:

1. Clinical practice reflect the fact that privacy is nuanced and HCPs can sometimes share genetic information without breaching confidentiality
2. Researchers define and make distinctions between levels of “genetic information” when seeking HCPs' and patients' views about privacy
3. HCPs factor into their considerations about disclosure the potential harm to family dynamics of nondisclosure and potential benefit of HCP disclosure, even when patients are withholding information
4. Researchers, policy-makers, and HCPs do not rely solely on published guidelines to help resolve disclosure dilemmas
5. Further research be conducted regarding practical ways for HCPs to share information, how HCPs can resolve competing interests within potentially dysfunctional families,^52,53^ and attitudes toward sharing information with family members in the context of genomic medicine

## Conclusions

In this synthesis, we have shown that, although HCPs generally felt a responsibility toward relatives, concerns including privacy and family dynamics prevented them from acting on this responsibility. Yet we also showed that HCPs can sometimes share information without intruding on privacy and that sharing might facilitate family dynamics. We argue that norms in genetic medicine warrant reconsideration, especially as more genome tests are used. We thus call for more nuanced research about familial and individual approaches that facilitates the clinical integration of genomic health care to enhance familial, not just individual, benefit.

## Disclosure

The authors declare no conflict of interest.

## Figures and Tables

**Table 1:**
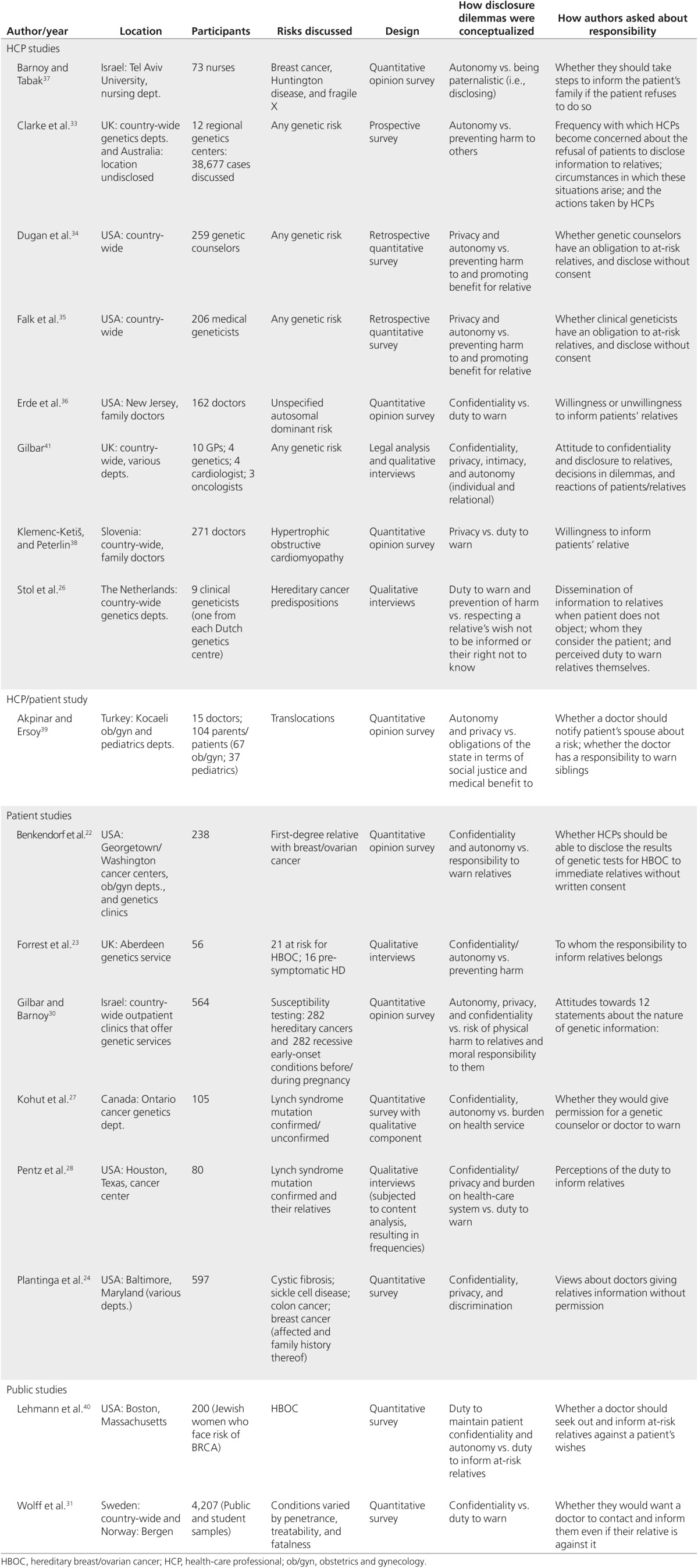
Detail of included studies

**Table 2:**
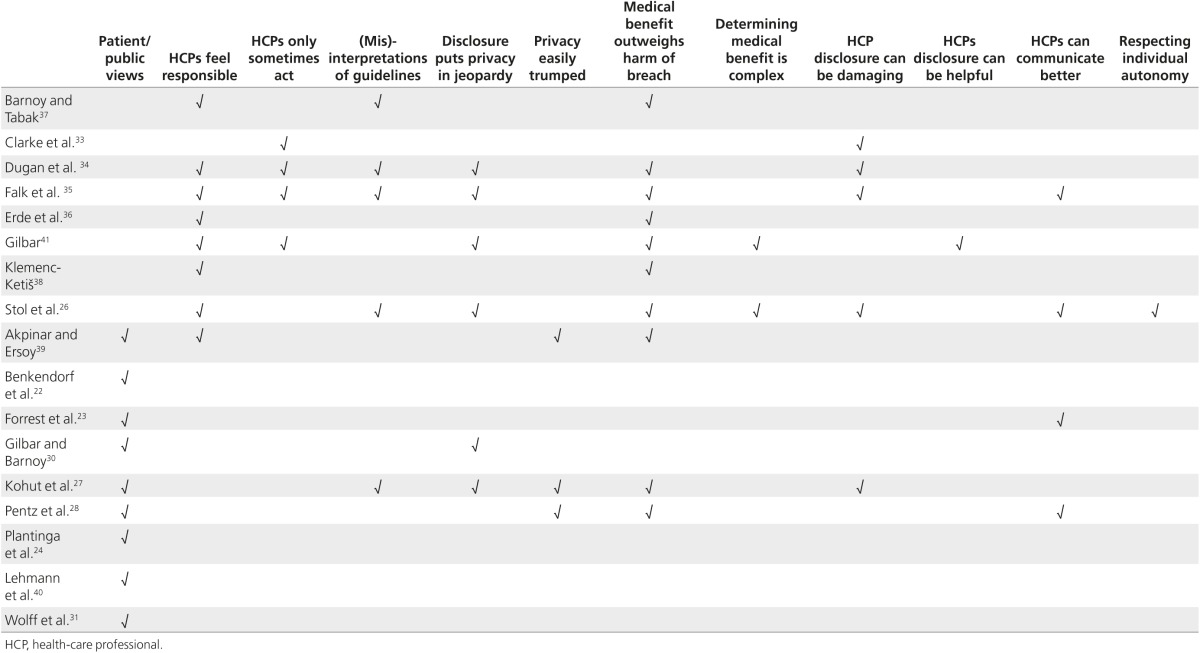
Topics discussed in each study
