# Supplementary material for: Health-care professionals' responsibility to patients' relatives in genetic medicine: a systematic review and synthesis of empirical research
Source: Genet Med. 2015 Jun 25;18(4):290–301. doi: 10.1038/gim.2015.72 (PMC4823639; doi:10.1038/gim.2015.72)
Supplement: Supplementary Table S1 [file gim201572x1.doc]

| Table S1: International guidelines/legislation about disclosure without consent | | |
| --- | --- | --- |
| Country | Guidelines/legislation | Detail |
| Australia | Privacy amendment (enhancing privacy protection) act 2012^1^ | Disclosure to relatives without consent is permissible if there is a serious threat to a relative’s life, health, or safety, and disclosure is necessary to lessen or prevent the threat. |
| Israel  (legislation) | Genetic information act (2000)^2,3^ | Only a hospital ethics committee can make the decision that confidentiality should be breached. Communication of the genetic information must be the only way of maintaining or improving the health of a relative (including an unborn relative), or prevent death, illness, or serious disability. The committee must deem the patient’s reasons for not sharing unreasonable, or the benefit to relatives as greater than the harm to the patient. |
| Netherlands | Dutch Society for Clinical Genetics (2007) Hereditary colorectal cancer guidelines/ hereditary breast and ovarian cancer guidelines^4^ | Family members of patients with hereditary breast and colorectal cancer should be informed about the options available for early diagnosis and treatment if there is a high probability of harm that could be avoided through receiving the information. |
|  | Dutch Society  for Clinical Genetics (2007) Predictive DNA research guidelines^4^ | If a patient indicates that they are not willing to cooperate in informing family members, the healthcare professional may decide to inform relatives against the patient’s wishes. |
| Norway (legislation) | Biotechological act (2003)^5^ | Patients decide whether to inform at-risk relatives of the condition. If a patient cannot or does not wish to inform at-risk relatives, healthcare professionals can request patient’s consent to inform relatives on their behalf. |
| Slovenia | Eurogentest (2011) Genetic testing guidelines^6^ | No specific guidance found. The European Society for Human Genetics (of which the Slovenian Society is a member) states where patient non-disclosure would cause serious harm, confidentiality can be breached. |
| Sweden  (legislation) | Genetic integrity act (2006)^5^ | The law does not forbid healthcare professionals from sharing information. However, it emphasises patients’ rights to self-determination and non-disclosure of genetic information, and urges healthcare professionals to consider the autonomy and integrity of the individual patient, even in cases of severe and inherited disorders involving other family members. |
| Turkey | Regulation on the  centres for diagnosis of genetic diseases (1998)^7^ | Genetic test results cannot be revealed to third parties without the consent of the patient. |
|  | Turkish Medical Association (2012) Code of medical ethics^7^ | The obligation of confidentiality becomes invalid in circumstances where keeping confidentiality would put the life of the patient or other people in jeopardy. |
| UK | General Medical Council (2009) Confidentiality guidelines^8^ | Sharing information without consent can be justified if it would benefit the relative and protect them from serious harm. If practicable, healthcare professionals should not disclose the patient’s identity when contacting and advising others of the risks they face. |
|  | British Society for Genetic Medicine/Joint Committee on Genomics in Medicine (2011) Consent and confidentiality guidelines^9^ | Healthcare professionals should err on the side of disclosing necessary de-identified information (e.g. a mutation report), rather than maintaining absolute confidentiality. |
| USA | American Society for Human Genetics (1998) Professional disclosure of familial genetic information guidelines^10^ | Information can be shared without consent if the patient persistently refuses to disclose to at-risk relatives; harm to at-risk relatives is highly likely, serious, imminent and foreseeable; the at-risk relative is identifiable; the disease is preventable or treatable or has early monitoring that could reduce risk; and the harm of non-disclosure is greater than the harm of the information being shared. |
| Canada | Covered by the ASHG | As above |

1. Government of Australia (2014) Use and disclosure of genetic information to a patient's genetic relatives under section 95AA of the Privacy Act of 1988 (Ch): guidelines for health practitioners in the private sector. Retrieved 20/04/15 from <https://www.nhmrc.gov.au/guidelines-publications/pr3>
2. Barnoy S, Tabak N. Israeli nurses and genetic information disclosure. Nurs Ethics. 2007;14(3):280-294.
3. The Israel Government (2000) Genetic Information Law, 5761-2000.
4. Stol YH, Menko FH, Westerman MJ, Janssens RM. Informing family members about a hereditary predisposition to cancer: attitudes and practices among clinical geneticists. J. Med Ethics. 2010;36(7):391-395.8
5. Eurogentest. Summary of the guidelines for genetic counselling. Retrieved 20/04/15 from http://www.eurogentest.org/index.php?id=675
6. Soini S. Genetic testing legislation in Western Europe—a fluctuating regulatory target. J Comm Genet. 2012;3(2):143-153.
7. Akpinar A, Ersoy N. Attitudes of physicians and patients towards disclosure of genetic information to spouse and first-degree relatives: a case study from Turkey. BMC Med Ethics. 2014;15(1):39.
8. General Medical Council (2009) Confidentiality. General Medical Council, London
9. British Society for Genetic Medicine / Joint Committee on Genomics in Medicine (2011) Consent and confidentiality in clinical genetic practice: Guidance on genetic testing and sharing genetic information. 2nd edn. BSGM, London
10. American Society for Human Genetics. Social Issues Subcommittee on Familial Disclosure (1998) Professional disclosure of familial genetic information. Am J Hum Genet 62(2):474–483
